# P-213. The Influence of National Population Composition Disparity on National Age–Adjusted Mortality and Disease Prevalence

**DOI:** 10.1093/ofid/ofaf695.435

**Published:** 2026-01-11

**Authors:** Soichi Takeishi, Tatsuo Inoue, Koichi Miyamura

**Affiliations:** Inuyama Chuo General Hospital, Inuyama-city, Aichi, Japan; Inuyama Chuo General Hospital, Inuyama-city, Aichi, Japan; Inuyama Chuo General Hospital, Inuyama-city, Aichi, Japan

## Abstract

**Background:**

We examined the effect of national population composition disparity on national age–adjusted mortality and disease prevalence.

**Figure:**
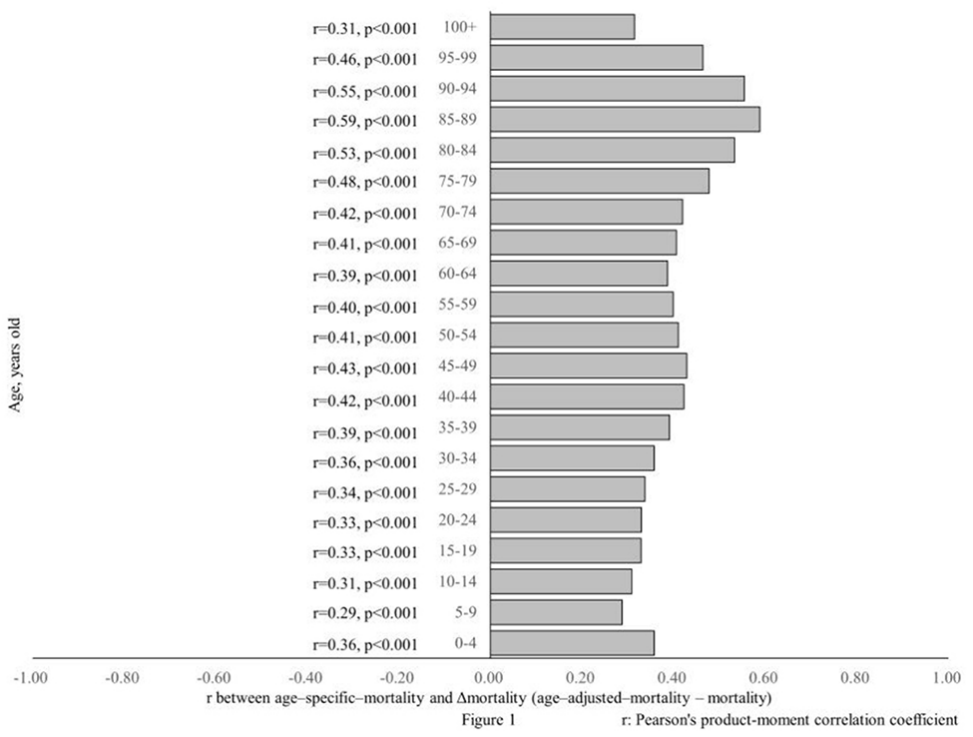

**Figure:**
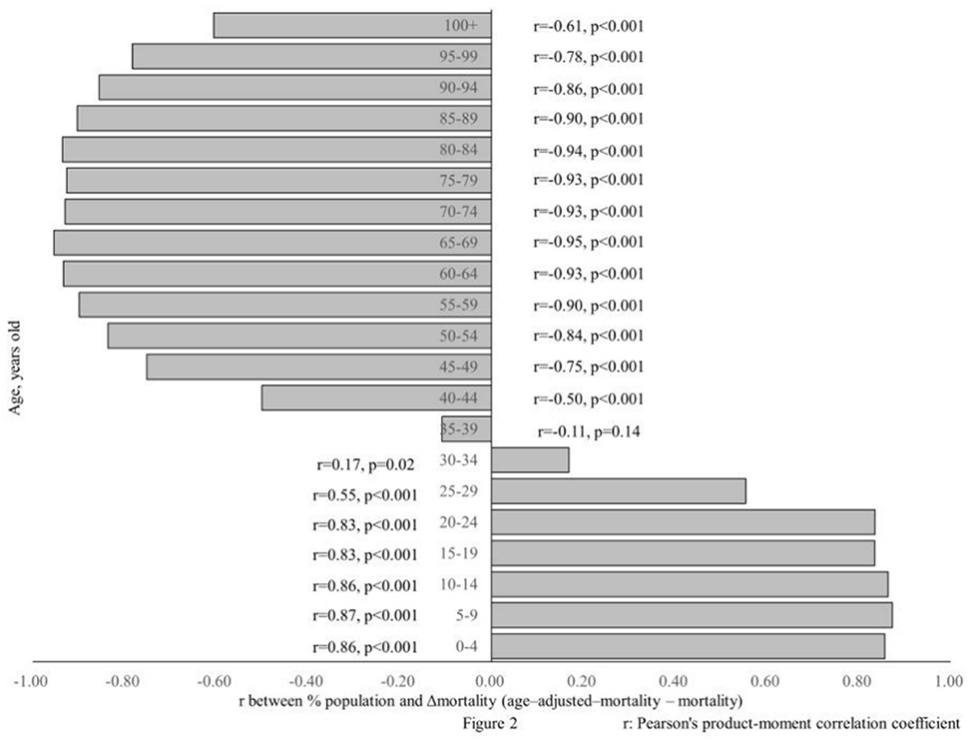

**Methods:**

For each “country or territory” (country), we proposed and calculated a metric named “% population” as follows: population for each 5–year age group ÷ population (0–100 years) × 100 (%), using United Nations World Population Prospects (WPP) 2019. We used age-adjusted diabetes prevalence (20–79 years) from the latest IDF Diabetes Atlas 2021. The diabetes prevalence was calculated using the latest IDF Diabetes Atlas 2021 as follows: the number of people with diabetes (20–79 years) ÷ population (20–79 years) × 100 (%). We used the latest number of deaths (0–100 years) from the WPP 2022. We calculated age–adjusted mortality (0–100 years) and mortality (0–100 years), using “population (0–100 years) from WPP 2019 + the number of deaths (0–100 years) from WPP 2022” as “Total population” (0–100 years) to ensure a consistent % population for both mortality and diabetes prevalence. Age–specific mortality was calculated as follows: the number of deaths for each 5–year age group ÷ “Total population” for each 5–year age group × 100 (%). Δrank mortality was defined as the “rank of descending order of age-adjusted-mortality in all countries – rank of descending order of mortality in all countries”. Similarly, Δrank prevalence was defined by replacing mortality with diabetes prevalence. Countries for which the required data could not be obtained were excluded.

**Figure:**
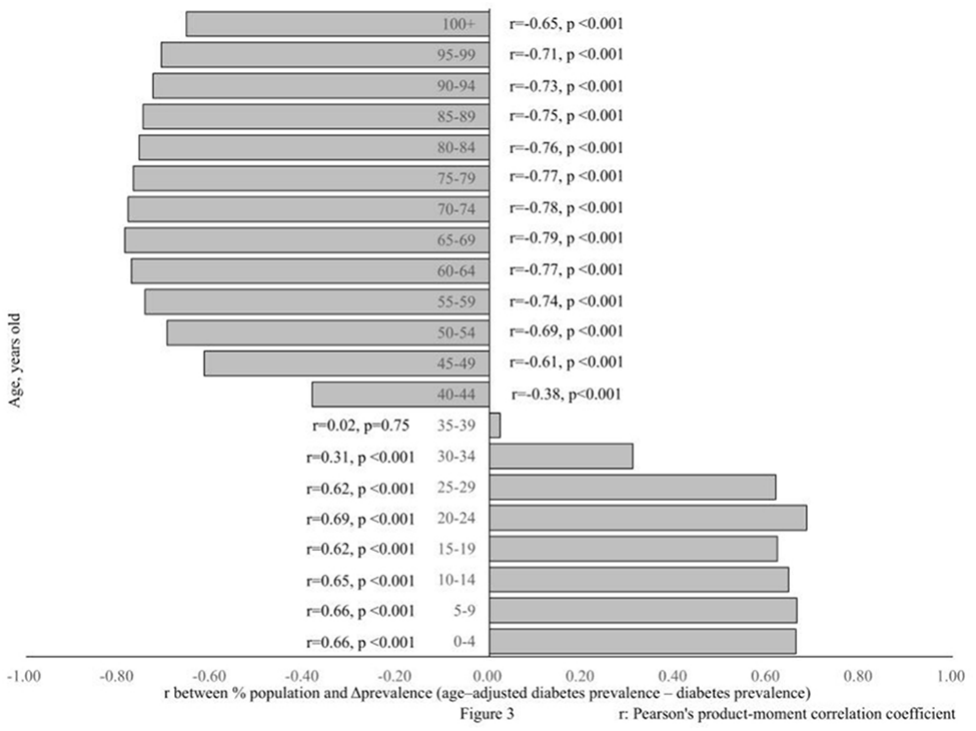

**Figure:**
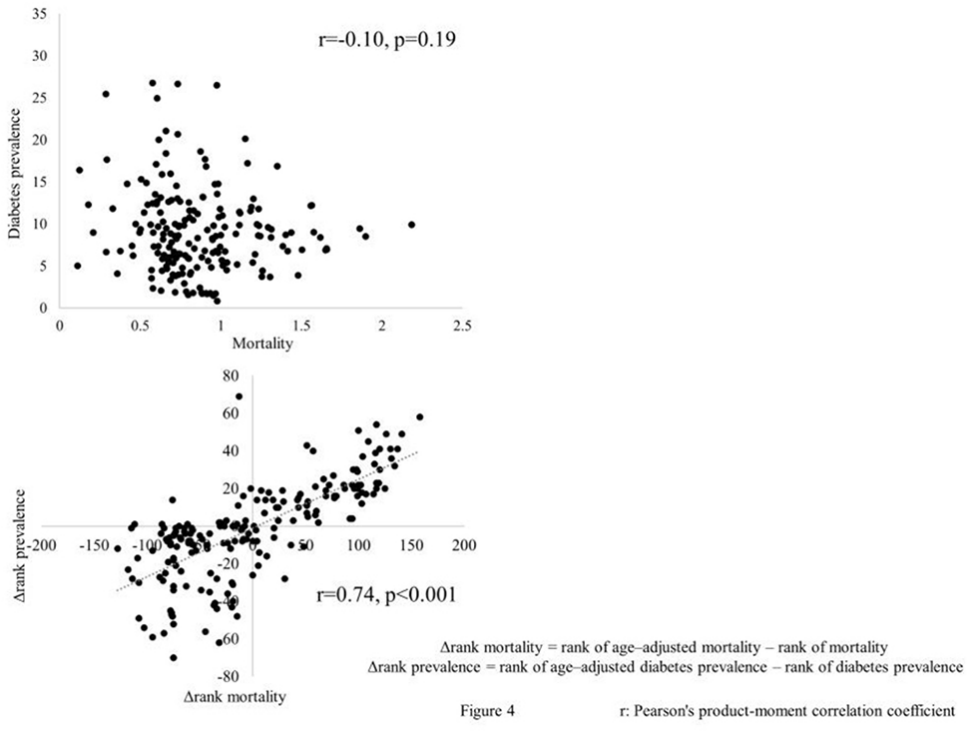

**Results:**

This study included 188 countries. For all 5–year age group, age–specific mortality was positively correlated with “age–adjusted mortality – mortality” (Δmortality) (mean r = 0.4) (Figure 1). For any 5–year age group younger than 35 years, % population was positively correlated with Δmortality (mean r = 0.61) and “age–adjusted diabetes prevalence – diabetes prevalence” (Δprevalence). For any 5-year age group older than 40 years, % population was negatively correlated with Δmortality (mean r = –0.83) and Δprevalence (Figures 2 and 3). Mortality did not correlate with diabetes prevalence. Δrank mortality was positively correlated with Δrank prevalence (Figure 4).

**Conclusion:**

National population composition disparity worldwide may significantly affect age adjustment.

**Disclosures:**

All Authors: No reported disclosures

